# Discourse-mediation of the mapping between language and the visual world: Eye movements and mental representation

**DOI:** 10.1016/j.cognition.2008.12.005

**Published:** 2009-04

**Authors:** Gerry T.M. Altmann, Yuki Kamide

**Affiliations:** a Department of Psychology, University of York, Heslington, York YO10 5DD, UK; b School of Psychology, University of Dundee, UK

**Keywords:** Sentence comprehension, Eye movements, Visual scene interpretation, Situation models

## Abstract

Two experiments explored the mapping between language and mental representations of visual scenes. In both experiments, participants viewed, for example, a scene depicting a woman, a wine glass and bottle on the floor, an empty table, and various other objects. In Experiment 1, participants concurrently heard either ‘The *woman will put the glass on the table*’ or ‘The *woman is too lazy to put the glass on the table*’. Subsequently, with the scene unchanged, participants heard that the woman ‘*will pick up the bottle, and pour the wine carefully into the glass.*’ Experiment 2 was identical except that the scene was removed before the onset of the spoken language. In both cases, eye movements after ‘*pour*’ (anticipating the glass) and at ‘*glass*’ reflected the language-determined position of the glass, as either on the floor, or moved onto the table, even though the concurrent (Experiment 1) or prior (Experiment 2) scene showed the glass in its unmoved position on the floor. Language-mediated eye movements thus reflect the real-time mapping of language onto dynamically updateable event-based representations of concurrently or previously seen objects (and their locations).

## 1 Introduction

Increasing attention has focused in recent years on language-mediated eye movements and the ‘visual world’ paradigm (Cooper, 1974; Tanenhaus, Spivey-Knowlton, Eberhard, & Sedivy, 1995). Language-mediated eye movements are commonly taken to reflect the cognitive processes that underpin the real-time processing of language, and the mapping of that language onto a concurrent visual world. Various studies have now shown the sensitivity of language-mediated eye movements to factors implicated in a wide range of phenomena associated with language comprehension (for reviews, see Henderson & Ferreira, 2004, and *Journal of Memory and Language, Vol. 57*). Typically, such studies measure eye movements around *static* scenes; for example, monitoring the likelihood with which certain objects depicted within the scene are fixated as a word or sentence unfolds. Recently, Hoover and Richardson (2008) and Knoeferle and Crocker (2007) explored the mapping of eye movements onto events depicted across either dynamically changing animations or multiple frames. In these studies, the eye movements of interest were towards a static, unchanging, scene that followed the animation or the multiple frames. Both studies showed that language-mediated eye movements reflect the mapping from language onto dynamically updateable mental representations of the visual ‘situation’, as distinct from the mapping of language onto static representations of the concurrent visual scene. But whereas these two studies were based on updating of the situation on the basis of spatiotemporal information afforded by the animations or multiple frames, the studies we report below show such updating on the basis of linguistic information alone. Moreover, we show (in Experiment 2) that such updating can occur, and more significantly, can drive eye movements, even when the visual scene has been removed prior to the onset of that linguistic information. These data will allow us to make a distinction between the representations of objects as depicted within a (concurrent or previous) visual scene and the representations of objects (and their locations) as maintained within the unfolding conceptual correlates of the event which the unfolding language describes.

Events have internal complexity, entailing, at a minimum, an initial state and an end state (with one or more participants in the event undergoing some change between the initial and end states); cf. Dowty (1979). Sentence comprehension in the context of a concurrent (but static) visual scene requires the comprehender to determine whether the scene corresponds to the initial state, the end state, or some intermediate state in the event described by the unfolding language (cf. Altmann & Kamide, 2007); thus, and notwithstanding the static nature of the concurrent visual depiction of the participants, it also requires the comprehender to keep track of changes to the participants in the event as the event, as described by the language, unfolds. Our interest here is in respect of the consequences for the cognitive mechanisms that might instantiate event representations, and the manifestation of these consequences on language-mediated eye movements, of the need to keep track of multiple representations of the *same* participant, albeit at different stages of the event. We shall argue below that these dynamically changing mental representations of the participants can, in certain circumstances, *compete* with their visually depicted counterparts.

The goal of much work within the visual world paradigm has been to investigate how (and when) the language that we hear makes contact with the world that we see. The evidence to date suggests that certain aspects of the language make contact with the visual world at the theoretically earliest opportunity. Work by Allopenna, Magnuson, and Tanenhaus (1998) and by Dahan and colleagues (e.g. Dahan, Magnuson, Tanenhaus, & Hogan, 2001) has shown how acoustic mismatch mediates looks towards potential referents at the earliest moments. Kamide, Altmann, and Haywood (2003) showed in addition how multiple information sources conspire to drive the eyes towards particular objects as soon as that information becomes available to the comprehender. They presented participants with a visual scene depicting a man, a child (a girl), a motorbike, a fairground carousel and various other objects. Concurrently, participants heard either ‘*The man will ride the motorbike*’ or ‘*The girl will ride the carousel*’. It was more plausible for the man to ride the motorbike than the carousel, and for the child to ride the carousel than the motorbike. During the acoustic lifetime of ‘*ride*’, more *anticipatory* looks were directed towards the motorbike when the subject was ‘*the man*’ than when it was ‘*the child*’. Conversely, more looks were directed towards the carousel during ‘*ride*’ after ‘*the child*’ than after ‘*the man*’. This result demonstrates that the eyes were directed towards distinct objects in the scene, during the acoustic lifetime of the verb, on the basis of the rapid integration of the meaning of the verb ‘*ride*’, the meaning of its grammatical subject (*‘the man*’ or ‘*the child*’), and the plausibility of the denoted event given the objects in the concurrent visual scene (that is, who, given the scene, would do the riding, and what, given that same scene, would most plausibly be ridden).

Kamide et al. (2003) suggested that their data are the hallmark of an incremental language processor that attempts at each moment in time to construct the fullest possible interpretation of the linguistic input (a claim that needs to be moderated to take account of goal-related factors that may lead to less than the fullest possible interpretation – cf. Ferreira, Ferraro, & Bailey, 2002). Importantly, language-mediated eye movements do not reflect only the workings of the language system. They reflect also the workings of whichever parts of the cognitive system interpret the external world. Indeed, Kamide et al. (2003) pointed out that their data were compatible with a hypothesis in which the assignment of thematic roles to objects in the visual world was as much driven by processes operating in the linguistic domain as by processes operating in the visual domain (see also Altmann & Kamide, 2004). They proposed that processes that are not language-specific, but which draw on experiential knowledge of objects and their interactions (i.e. *affordances*), establish thematic relations between objects in the scene, both in relation to each other and in relation to the thematic roles which the unfolding language may make available (cf. Chambers, Tanenhaus, & Magnuson, 2004; Knoeferle & Crocker, 2006; Knoeferle, Crocker, Scheepers, & Pickering, 2005). This view, that ‘situated’ sentence interpretation draws on domain-independent processes operating over experiential and situational knowledge, is little different to that expressed by Zwaan and Radvansky (1998), who review a range of studies suggesting that modality-independent processes are used to construct situation models during reading, listening, and viewing. In the studies we report below, we consider how the construction and dynamic modification of such situation models impacts on language-mediated eye movements; we show how discourse context can, by dynamically changing the situation, change aspects of the mental representations of the objects depicted within a concurrent but unchanging scene (in fact, those aspects to do with the distinct locations that an object moves through as the situation/event unfolds); we show that such changes mediate subsequent eye movements towards the scene as the language unfolds. Thus, we shall demonstrate how language is mapped not onto static representations of the (static) scene, but rather onto dynamically changing (and changeable) representations of that scene.

Our starting point for this work can be exemplified by the following: ‘*Paul will take the watch off his wrist, and place it around Jeanne’s wrist*’. Our mental representation of the events denoted by this sentence include the initial state (the watch on the wrist), intermediate states (entailed by the action associated with taking off the watch), and the end state (the watch on Jeanne’s wrist). Each of these states must be encoded with respect to some internalized time line (without which the causal relationships between these states and the events denoted by the sentence would not be apparent). If this sentence were to be followed by ‘*She will admire the watch for a moment, before removing it*’ we would interpret ‘*the watch’* to mean the watch *after* it has been taken off Paul’s wrist and placed on her own wrist. On the other hand, the first mention of the watch in ‘*Paul will take the watch off his wrist*’ is taken to refer to the watch on his wrist. So what are the consequences, for our interpretation of the final token of *‘the watch*’, if Paul is standing before us with his wristwatch plainly in view, on his wrist, and as yet unmoved? How, if at all, does the cognitive system keep apart the representation of the concurrent watch (on Paul’s wrist) and the representation of this same watch at some distinct time (in the future) and location (on Jeanne’s wrist)? Presumably, the processing system uses information about, in this case, the tense of the sentence to indicate a future timeframe, and on this basis the representations can be referred to with little confusion. We have elsewhere established that tense information is indeed used, as a sentence unfolds in real-time, to constrain which representations are being referred to (Altmann & Kamide, 2007; cf. Knoeferle & Crocker, 2007). The experiments below attempt instead to establish the *attentional consequences* of referring to something that is co-present in the visual world but which is referred to in the context of some future change in location of that object. In effect, we ask here what happens when people see Paul and the watch upon his wrist, and Jeanne (watchless), and hear the sequence above (about Paul taking off his watch and placing it around Jeanne’s wrist) and hear mention of the watch – if hearing mention of the watch when it refers to the watch on his wrist causes the eyes to move towards his wrist as the word ‘*watch*’ unfolds (cf. Allopenna et al., 1998; Cooper, 1974), where will the eyes move when ‘*the watch*’ refers to that ‘version’ of the same watch when it is on Jeanne’s wrist? Will they look towards the ‘original’ (and co-present) watch, or to the location of this future version of the watch (i.e. her wrist)? On the assumption that the representation of the watch *as encoded from the language* (i.e. the watch that is no longer on Paul’s wrist) contains information pertaining to the watch’s new location (i.e. as being on Jeanne’s wrist), can this information mediate visual attention during subsequent reference to the watch?

## 2 Experiment 1

**Fig. 1:**
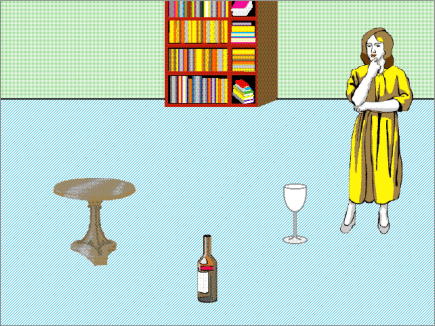
Example scene from Experiments 1 and 2. See the main text for the accompanying sentential stimuli.

The purpose of this study was to determine whether eye movements are mediated by the content of a concurrent visual scene, or by the content of mental representations that have an existence that is, at least in part, dissociable from the perceptual correlates of the objects in that scene. We manipulated the mental representations by presenting a contextualizing sentence which described how one of the objects in the concurrent scene was going to be moved by a protagonist (also depicted in the scene) to a new location. For example, the scene shown in Fig. 1 depicts, amongst other things, a woman, an empty wine glass, and a table. Immediately prior to a target sentence that referred to the woman pouring the wine into the glass, we presented first either ‘*The woman will put the glass on the table*’ or ‘*The woman is too lazy to put the glass onto the table*’. Both the context sentence and the subsequent target sentence were presented concurrently with the visual scene, but the first of the two alternative context sentences changed the *mental location* of the glass from the floor to the table. The second of the two alternatives left the location unchanged.

In this study, we shall ask two questions of the data: First, where do participants’ eyes move as they *anticipate* the most plausible location of the pouring? Kamide et al. (2003); Experiment 1 observed anticipatory eye movements towards the most plausible goal object following a ditransitive verb such as ‘pour’ (i.e. towards the receptacle) during the post verbal region of the sentence. We shall take advantage of this phenomenon to establish where the interpretive systems assumes that goal object to be – will the eyes move more towards the table in the ‘moved’ condition than in the ‘unmoved’ (‘*too lazy*’) condition? Or will they move towards the glass irrespective of the prior context? Second, we shall ask where will participants’ eyes be looking *after* they have heard ‘*glass*’ in the target fragment ‘*she will pick up the bottle and pour the wine carefully* *into the glass*’ – will looks at the physical location of the glass be unaffected by the prior context? Or might its ‘mental location’, as determined by the prior context, determine the likelihood of looking at the glass or table?

### 2.1 Method

#### 2.1.1 Subjects

Thirty-two participants from the University of York student community took part in this study. They participated either for course credit or for £2.00. All were native speakers of English and either had uncorrected vision or wore soft contact lenses or spectacles.

#### 2.1.2 Stimuli

Sixteen experimental pictures (see Fig. 1) were paired with two sentential conditions corresponding to (1) and (2) below.

(1) The woman will put the glass onto the table. Then, she will pick up the bottle, and pour the wine carefully into the glass.
(2) The woman is too lazy to put the glass onto the table. Instead, she will pick up the bottle, and pour the wine carefully into the glass.

The first sentence in each condition always referred to the agent, the theme, and the goal; the two conditions were designed to be minimally different. See Appendix 1.

The visual scenes were created using commercially available ClipArt packages, and were constructed using a 16-colour palette. The scenes corresponding to each experimental item are described in Appendix 1. They were presented on a 17” viewing monitor at a resolution of 640 × 480 pixels. The same target sentence was used across both context conditions. A further 32 sentence/scene pairs were added as fillers. These employed similar pictures to the experimental items but included a range of other sentence types. The materials were arranged in a fixed-random order so that no successive items belonged to the same condition. Two lists of stimuli were created containing each of the 16 experimental pictures but just one version of each sentence pair. The sentences were recorded by a male native speaker of British English (GTMA), and sampled at 44.1 KHz. The sound files were presented to participants via a mono channel split to two loudspeakers positioned either side of the viewing monitor. The onsets and/or offsets of critical words in the stimulus sentences were marked using a sound editing package for later analysis.

#### 2.1.3 Procedure

Participants were seated in front of a 17” display with their eyes approximately 60 cm. from the display. They wore an SMI EyeLink head-mounted eye-tracker, sampling at 250 Hz from the right eye (viewing was binocular). Participants were told that they would be shown some pictures that would be accompanied by a short sentence spoken over loudspeakers. In respect of their task, participants were simply told that ‘*we are interested in what happens when people look at these pictures while listening to sentences that describe something that might happen in the picture*’ (see Altmann, 2004 for discussion of the task). Between each trial, participants were shown a single centrally-located dot to allow for any drift in the eye-track calibration to be corrected. This dot was then replaced by a fixation cross and participants would press a response button for the next trial. The onset of the visual stimulus preceded the onset of the spoken stimulus by 1000 ms. The trial was automatically terminated after 10 or 12 s, depending on the length of the auditory stimulus. After every fourth trial, the eye-tracker was recalibrated using a 9-point fixation stimulus. Calibration took approximately 20 s. There were four practice trials before the main experimental block. The entire experiment lasted approximately 25 m.

### 2.2 Results

Eye movements that landed more than one or two pixels beyond the boundaries of the target object were not counted as fixations on that object. As in previous studies (e.g. Altmann & Kamide, 2007) we make no claims here regarding either the resolution of the eye tracker or the accuracy of eye movements. We adopt this criterion simply to avoid having to make a potentially arbitrary decision regarding how far a fixation can land from an object but still be deemed to have been directed towards that object. We report in Table 1 four eye movement measures synchronized on a trial-by-trial basis with the target sentence. For the sake of exposition, we shall refer to the target with the example ‘*she will pick up the bottle, and pour the wine carefully into the glass*’. The first measure we report is the probability of fixating the glass or the table at the onset of ‘*the wine*’ (i.e. at the onset of the determiner). At this point in the sentence, we do not anticipate any bias to look towards one or other object (participants at this stage are most likely anticipating the theme, not the goal – Kamide et al., 2003, observed anticipatory eye movements in equivalent ditransitive constructions towards the glass *during* ‘*the wine*’, but not before). The second measure is the probability of launching at least one eye movement towards the glass or table during the underlined portion of ‘*she will pick up the bottle, and pour the wine carefully into the glass*’ (i.e. in the region between the onset of the postverbal determiner and the onset of the final determiner). We report also the probability of launching at least one eye movement towards the glass or table during the final noun phrase ‘*the glass*’ and, finally, the probability of fixating the glass or table at the offset of ‘*glass*’. We include these last three measures in order to establish, first, where participants looked when *anticipating* the goal location of the pouring (cf. Altmann & Kamide, 1999; Kamide et al., 2003), and second, where participants were looking once they knew, on the basis of the acoustic input, that the glass was indeed the intended goal of the pouring. Probabilities were calculated by summing, for saccades, the number of trials in which at least one saccadic eye movement was directed towards the target during the critical region, or for fixations, the number of trials in which the target was fixated at the onset of the critical word. In each case, we took into account on a trial-by-trial basis the actual onsets/offsets of the critical words in the auditory stimulus.

Statistical analyses were performed using hierarchical log-linear models. Log-linear models are appropriate in the analysis of frequency or probability data, which are necessarily bounded. The equivalent of planned comparisons were computed by establishing an interaction (or lack thereof) between condition (‘moved’ vs. ‘unmoved’) and object (e.g. the table vs. all other objects, or the glass vs. all other objects). This analysis allows us to take into account the necessary dependency on each trial between looks to one object and looks to all others. If an effect of context is found on both the table and the glass, we need then to establish whether these effects are carried solely by the table and the glass (i.e. looks towards the table being *at the expense* of looks towards the glass, and vice versa), or whether they might be carried by (unexplained) looks elsewhere in the scene. To do this, we contrast looks towards the table *and* the glass (taken together) with looks elsewhere. Failing to find an interaction between condition (‘moved’ vs. ‘unmoved’) and object (the table and the glass vs. the rest of the scene) would establish that the number of looks towards the table and the glass, taken together, were constant across condition, meaning in turn that the effects of context on the glass were complementary to the effects of context on the table (see Altmann & Kamide, 2007, for discussion of this logic). Participants and items were entered, separately, as factors in the computation of partial association Likelihood Ratio Chi-Squares (LRCS_1_ and LRCS_2_, respectively) in order to assess the generalizability of the effects across participants and items.^1^ For further discussion of the use of log-linear models in this experimental paradigm, see Altmann and Kamide (2007), Huettig and Altmann (2005), and Scheepers (2003).

(i). *At the onset of ‘the wine’:* The probability of fixating the table was marginally greater in the ‘moved’ condition than in the ‘unmoved’ condition (LRCS_1_ = 3.9, df = 1, *p* = .049; LRCS_2_ = 3.5, df = 1, *p* = .062), consistent by participants and by items (LRCS_1_ = 22.9, df = 31, *p* > .8; LRCS_2_ = 19.6, df = 15, *p* > .1). The probability of fixating the glass did not vary by condition (LRCS_1_ = 0, df = 1, *p* = 1; LRCS_2_ = 0.02, df = 1, *p* > .8), consistent by participants and by items (LRCS_1_ = 35.6, df = 31, *p* > .2; LRCS_2_ = 16.7, df = 15, *p* > .3). Overall, the glass was fixated significantly more often than the table (LRCS = 7.1, df = 1, p < .01), consistent by participants and by items (LRCS_1_ = 23.1, df = 31, p > .8; LRCS_2_ = 14.4, df = 15, p > .4).
(ii). *During ‘the wine carefully into’:* The probability of launching an anticipatory eye movement towards the table was significantly higher in the ‘moved’ condition than in the ‘unmoved’ condition (LRCS_1_ = 15.9, df = 1, *p* < .001; LRCS_2_ = 13.5, df = 1, *p* < .001), consistent by participants and by items (LRCS_1_ = 30.9, df = 31, *p* > .4; LRCS_2_ = 14.7, df = 15, *p* > .4). The probability of launching an eye movement towards the glass did not differ, statistically, between the two conditions (LRCS_1_ = 1.8, df = 1, *p* > .1; LRCS_2_ = 2.2, df = 1, *p* > .1), consistent by participants and by items (LRCS_1_ = 17.9, df = 31, *p* > .9; LRCS_2_ = 14.6, df = 15, *p* > .4). Saccades were launched more often towards the glass than towards the table (LRCS = 49.1, df = 1, *p* < .001), consistent by participants and by items (LRCS_1_ = 31.6, df = 31, *p* > .4; LRCS_2_ = 14.3, df = 15, *p* > .5).
(iii). *During ‘the glass’:* The probability of launching an eye movement towards the table was significantly higher in the ‘moved’ condition than in the ‘unmoved’ condition (LRCS_1_ = 12.2, df = 1, *p* < .001; LRCS_2_ = 13.0, df = 1, *p* < .001), consistent by participants and by items (LRCS_1_ = 31.6, df = 31, *p* > .4; LRCS_2_ = 21.8, df = 15, *p* > .1). The probability of launching an eye movement towards the glass did not differ, statistically, between the two conditions (LRCS_1_ = 1.6, df = 1, *p* > .2; LRCS_2_ = 1.7, df = 1, *p* > .1), consistent by participants and by items (LRCS_1_ = 26.5, df = 31, *p* > .6; LRCS_2_ = 12.0, df = 15, *p* > .6). Saccades were again launched more often towards the glass than towards the table (LRCS = 37.6, df = 1, *p* < .001), consistent by participants and by items (LRCS_1_ = 23.3, df = 31, *p* > .8; LRCS_2_ = 23.4, df = 15, *p* > .07).
(iv). *At the offset of ‘the glass’:* the table was fixated more at this point in time in the ‘moved’ condition than in the ‘unmoved’ condition (LRCS_1_ = 14.3, df = 1, *p* < .001; LRCS_2_ = 13.0, df = 1, *p* < .001), consistent by participants and by items (LRCS_1_ = 33.8, df = 31, *p* > .3; LRCS_2_ = 17.4, df = 15, *p* > .2). And conversely, the glass was fixated more in the ‘unmoved’ condition than in the ‘moved’ condition, although the effect just failed to meet statistical significance in the by-items analysis (LRCS_1_ = 4.8, df = 1, *p* < .03; LRCS_2_ = 3.8, df = 1, *p* = .051), consistent by participants and by items (LRCS_1_ = 31.8, df = 31, *p* > .4; LRCS_2_ = 19.6, df = 15, *p* > .1). To the extent that there were effects of context on both fixations on the table and fixations on the glass, these effects were complementary – that is, increased fixations on the table were at the expense of decreased fixations on the glass, and vice versa (there was no effect of context on proportions of looks to non-critical regions (see above for the rationale for this analysis): LRCS_1_ = 0.03, df = 1, *p* > .8; LRCS_2_ = 0.08, df = 1, *p* > .7). This was consistent by items (LRCS_2_ = 20.3, df = 15, *p* > .1), but was not consistent by participants (LRCS_1_ = 51.4, df = 31, *p* < .02), meaning that the magnitude of the context × object (glass/table vs. distractors) interaction varied across participants (most likely, this should be interpreted as meaning that for some participants, the increased fixations on the table in the ‘moved’ condition were accompanied not only by decreased fixations on the glass, but also by decreased fixations on other regions of the scene; this increase in fixations on the table was overall slightly larger than the corresponding decrease in fixations on the glass and could not therefore be entirely accounted for by decreased fixations on the glass). There were, overall, more fixations on the glass than on the table (LRCS = 71.7, df = 1, *p* < .001), Saccades were launched more often towards the glass than towards the table (LRCS = 37.6, df = 1, *p* < .001), consistent by participants and by items (LRCS_1_ = 27.7, df = 31, *p* > .6; LRCS_2_ = 17.6, df = 15, *p* > .2).

**Fig. 2:**
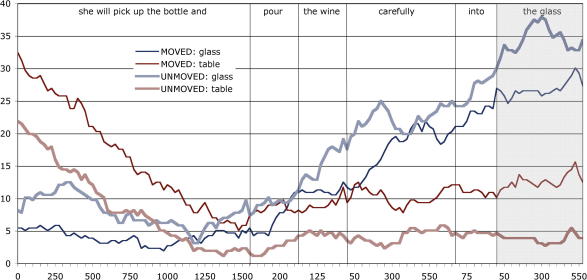
Percentage of trials in Experiment 1 with fixations on the regions of interest corresponding to the table and the glass in the ‘moved’ and ‘unmoved’ conditions during ‘*she will pick up the bottle and pour the wine carefully into the glass*’ or its equivalent across trials. The percentages reflect the proportion of trials on which each of the regions of interest was fixated at each moment in time, and were calculated at each successive 25 ms from the synchronization point. See the main text for a description of the resynchronization process. The region of the graph corresponding to the target noun phrase ‘*the glass*’ is highlighted.

In Fig. 2, we plot the percentage of trials with fixations on each of the two regions of interest (the table and the glass), in 25 ms. increments from the onset of, and during, ‘*the wine carefully into the glass*’. As noted in Altmann and Kamide (2004), there is an inherent problem in interpreting such plots; for example, ‘zeroing’ time at the onset of ‘*the wine carefully into the glass*’ results in a mean onset of ‘*the glass*’ at 1246 ms. However, this is just the average across stimuli (and hence, across trials), with a minimum onset at 1014 ms. and a maximum onset at 1510 ms. This renders the interpretation of such plots problematic; the further into the sentence fragment, the greater this degree of desynchronization between the unfolding speech and the unfolding eye movement plot. In order to avoid this cumulative desynchronization, the curves in Fig. 2 (and Fig. 4, below) are *resynchronized* at each of the points shown by the vertical lines (in effect, separate curves are calculated for each interval of interest, and ‘stitched’ together). Thus, instead of just one synchronization point at the onset of the sentence fragment, there are seven synchronization points (including fragment onset and fragment offset). This guarantees that the intersection between each curve and each vertical synchronization line accurately reflects the probability of fixation at the corresponding moments in time across all trials. Hence multiple zeros on the *x*-axis of each plot. A further problem with such plots, also described in Altmann and Kamide (2004), is that fixation plots do not accurately reflect the moment-by-moment shifts in overt attention that are accompanied by saccadic eye movements. Fixations and saccades can dissociate; a period of time in which the likelihood of fixation on a region is constant may also be a period of time in which the likelihood of a saccadic movement to that region rises (and conversely, saccadic movements *out* of that region also rise). This dissociation is task-dependent, and is less apparent for example in reaching tasks where the eye will tend to maintain fixation on the to-be-reached target. In the ‘look and listen’ task we employ here, the dissociation between fixations and saccades is more apparent; hence our reporting, and statistical analyses, across both fixational *and* saccadic measures. The graph in Fig. 2 (and Fig. 4 below) is thus provided for illustrative purposes only, with the data in Table 1 (and 2 below) reflecting more directly the statistical analyses of these two measures, and indeed, reflecting more directly shifts in overt attention towards the glass or the table.

### 2.3 Discussion

Participants were more likely to look, postverbally, towards the table in the ‘moved’ condition than in the ‘unmoved’ condition. Indeed, at the one point in the sentence when participants could be absolutely certain that the glass was the object under consideration, namely at the offset of the sentence-final ‘*glass*’, they ended up looking at the table *or* the glass as a function of the context; that is, as a function of where the glass was located in the contextually determined mental representation of the scene and the objects it contained. It would appear, therefore, that language-mediated eye movements can be driven by the mapping of a sentence onto the contents of a dynamically updateable situational model in which the locations of the objects can be dynamically updated; eye movements were driven by the encoded locations of those objects, rather than just their actual locations as determined by the concurrent image. This conclusion is subject to two caveats, however. First, there was no effect of context on looks towards the glass until the final point in the sentence; thus, increased looks towards the table before this point were not at the expense of fewer looks towards the glass (and moreover, even at the final point in the sentence, some caution should be exercised in respect of the effect of context on fixations on the glass, given that this effect was not entirely reliable, statistically, in the by-items analysis; *p* = .051). Second (but possibly related – see below), notwithstanding the effect of context on looks towards the table, there was always a preference to look towards the glass (see Table 1 – this preference could not be an artefact of differences in size, across trials, between the region corresponding to the glass and that corresponding to the table: the former was generally smaller than the latter). Thus, although (some) eye movements were driven by the encoded location of the glass, as either on the floor or on the table, this is not the whole story: they were only partly driven by the encoded locations, and were driven more by the actual location of the glass in the image. So how, then, can we reconcile the reliable effects of context on looks towards the table, when anticipating the glass or hearing ‘*the glass*’, with the overall preference to look towards the depicted glass?

One possibility is to take into account that participants had to keep track of multiple representational instantiations of the glass – the glass as depicted in the scene, and the glass as described by the unfolding language and which, at some future time, would be located on the table.^2^ Evidently, we do keep track of such multiple representational instantiations; otherwise, how else could we say to someone how sober (or not) they looked the night before without confusing the person in the here-and-now with the person as they were in the there-and-then? Given this need to maintain multiple representations of the same object, indexed to different events and locations, it follows that in the two conditions of Experiment 1, there are multiple instantiations of the glass that must be kept apart: the glass depicted on the floor in the here-and-now, the glass filled at some later time with wine, and in the ‘moved’ condition, the glass located on the table at the time of the pouring of the wine. These distinct representations must be kept apart, and as such, may in fact *compete*. Thus, on hearing ‘*the glass*’ at the end of the final sentence in Experiment 1, the two different instantiations of the glass – one depicted concurrently in the scene, and the other referred to by the language – may each compete for attention. Given the salience of the currently depicted glass (given its concurrent physical/sensory correlates), we might suppose that it ‘wins out’ in respect of this competition, and hence the bias to look towards the concurrently depicted glass when anticipating the glass (even in the ‘moved’ condition). The fact that looks to the table were modulated by the context, and looks to the glass also (albeit manifesting only in fixations on the glass at the end of the sentence) suggests that the representational instantiation of the glass on the table did ‘attract’, or guide, looks to some extent, notwithstanding the preference for looks to be guided primarily by the depiction of the glass on the floor. In Experiment 2 we test this hypothesis by eliminating the concurrent glass from the scene, and thereby eliminating the competition between the stimulus-driven representation of that glass and the event-based representation of the glass as described by the unfolding language. We predict that by eliminating this competition, the overall bias to look towards the physical location of the glass will itself be eliminated.

## 3 Experiment 2

Experiment 2 is identical to Experiment 1 except that the scenes were *removed* before the onset of the spoken sentences. Altmann (2004) showed participants scenes depicting, for example, a man, a woman, a cake, and a newspaper. The scenes were removed after a few seconds, and shortly after, participants heard sentences such as ‘*The man will eat the cake*’ while the screen remained blank. It was found that the eyes nonetheless moved, during ‘*eat*’ in this example, towards where the cake *had been* (cf. Altmann & Kamide, 1999, who showed the equivalent effect when the scene and accompanying sentence were concurrent). The rationale for using this blank screen paradigm for Experiment 2 is as follows: once the scene has been removed, information about where the glass is located can be based on only two sources of information – the memory of where the glass had actually been located in the prior scene, and the event-representations constructed as the spoken sentences unfold. Both of these are internal representations that do not have any concurrent physical correlates (unlike the actually depicted glass in the scene; in that case, the internal representation corresponding to that glass does have concurrent physical correlates). Conceivably, the visual memory of where the glass had actually been located is a more salient representation of the location of the glass than the event-representations constructed through the language (it is grounded, after all, in prior physical correlates). However, if this visual memory constitutes a temporary record of the experience of the glass, including its location (cf. an ‘episodic trace’), then this memory (like the representations constructed by the unfolding language) is also event-based (cf. Hommel, Müsseler, Aschersleben, & Prinz, 2001; and affordance-based accounts of object representation; e.g. Gibson, 1977; see Steedman, 2002, for a formal treatment of affordances as event representations). If the representation corresponding to the visual memory of the glass is itself an event-based representation, then perhaps it is no more salient a representation than the event-based representations constructed through the language. Indeed, the latter must presumably act upon versions of the former (they cannot directly modify the episodic memory of the object or it would not be possible to keep apart the episodic memory from the language-cued event-representation that refers to the glass in an alternative location). Thus, the visual memory of where the glass had actually been located may be no more salient (i.e. may attract no more attention) than the language-induced representation of where it will be located (after the event described by the language has unfolded). Whether this is in fact the case is an empirical issue which Experiment 2 addresses, and we postpone further discussion of the relative saliency of these different representations until the discussion section below. Critically, our intention is to establish whether it was indeed the concurrent physical representation of the glass in Experiment 1 that caused the overall preference to look towards this glass even in the ‘moved’ condition.

### 3.1 Method

#### 3.1.1 Subjects

Thirty-four participants from the University of York student community took part in this study. They participated either for course credit or for £2.00. All were native speakers of English and either had uncorrected vision or wore soft contact lenses or spectacles.

#### 3.1.2 Stimuli

The visual and auditory stimuli were identical to those used in Experiment 1 except for the 24 fillers used in this study. These included stimuli for an unrelated blank screen study, but all the filler stimuli were similar in respect of the complexity of the scenes and associated sentence types).

#### 3.1.3 Procedure

The same procedure was employed as for Experiment 1 except that a 22” monitor was used and the scenes were presented for 5 s before being replaced by a light grey screen. The onset of the auditory stimulus (corresponding to the context and target sentences) occurred 1 s after the scene had been removed. Eye movements were monitored throughout using an EyeLink II head-mounted eye-tracker sampling at 250 Hz, and the trial terminated 11 s after the onset of the auditory stimulus. Thus, each trial lasted for 17 s in total.

### 3.2 Results

**Fig. 3:**
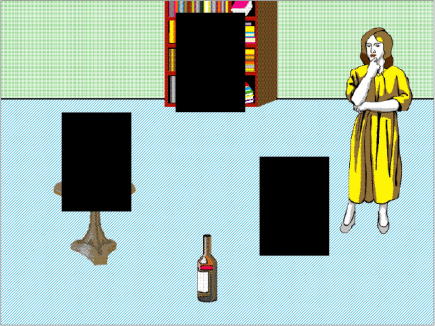
Example regions of interest, shown in black, for Experiment 2 superimposed over an example scene.

**Fig. 4:**
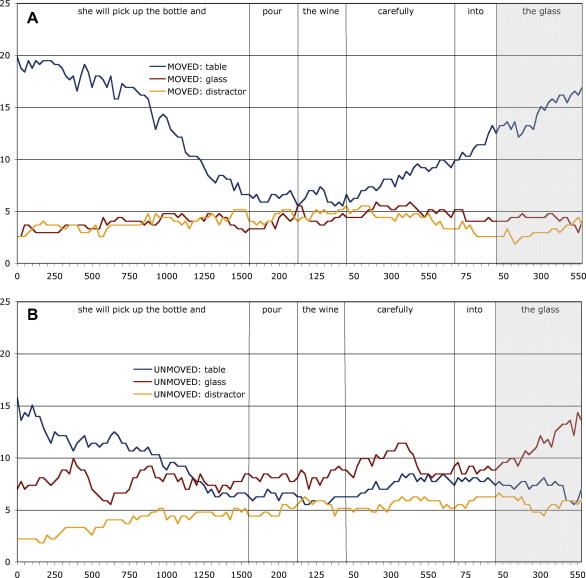
Percentage of trials in Experiment 2 with fixations on the regions of interest corresponding to where the table, glass, or distractor had been during ‘*she will pick up the bottle and pour the wine carefully into the glass*’ or its equivalent across trials. The percentages were calculated as for Experiment 1. Panel A shows the data from the ‘moved’ condition; Panel B shows the ‘unmoved’ data.

Whereas in Experiment 1 a fixation was deemed to have landed on an object if it fell on the pixels occupied by that object, we adopted a different scheme for defining regions of interest in this experiment. A rectangular box was drawn around either the location previously occupied by the glass or around the location to which it was ‘moved’ (in this case, a region encompassing the table top). The two rectangles, corresponding to where the glass or table top had been, were of identical size (although the size of these regions of interest varied on a trial-by-trial basis depending on the visual objects whose locations they indicated, but within each trial, the two regions of interest were identically sized). A third identically sized rectangle was placed at the location previously occupied by one of the distractor objects (e.g. the lower part of the bookshelf shown in Fig. 1). We included this region for the purpose of comparison with the other two regions (corresponding to where the glass or the table top had been), given that within the blank screen paradigm, comparison between different equally-sized regions can be made without the possibility that differences in looks might be due to differences in low-level visual salience (each region of interest is, after all, identical). Thus, any differences across condition can only be due to biases introduced by the mental representations constructed through the interplay between the unfolding language and the memory of what had previously occupied the scene. An example of these regions of interest, superimposed over the original image, is shown in Fig. 3. We report in Table 2 and Fig. 4 the same eye movement measures as were reported for Experiment 1, although in the present experiment, eye movements during the unfolding sentence were directed towards empty space. Of interest is *which* empty space the eyes were directed towards.

(i). *At the onset of ‘the wine’:* The probability of fixating where the table had been in the ‘moved’ condition was the same as that in the ‘unmoved’ condition (LRCS_1_ = 0.34, df = 1, *p* > .5; LRCS_2_ = 0.28, df = 1, *p* > .5), consistent by participants and by items (LRCS_1_ = 24.14, df = 33, *p* > .8; LRCS_2_ = 11.88, df = 15, *p* > .6). The probability of fixating where the glass had been also did not vary by condition (LRCS_1_ = 2.39, df = 1, *p* > .1; LRCS_2_ = 1.73, df = 1, *p* > .1), consistent by participants and by items (LRCS_1_ = 24.87, df = 33, *p* > .8; LRCS_2_ = 21.31, df = 15, *p* > .1). There were, overall, no more fixations on where the glass had been than on where the table had been (LRCS = 0.36, df = 1, *p* > .5), consistent by participants and by items (LRCS_1_ = 19.84, df = 33, *p* > .9; LRCS_2_ = 19.66, df = 15, *p* > .1).
(ii). *During ‘the wine carefully into’:* The probability of launching an anticipatory eye movement towards where the table had been was significantly higher in the ‘moved’ condition than in the ‘unmoved’ condition (LRCS_1_ = 10.0, df = 1, *p* < .002; LRCS_2_ = 7.37, df = 1, *p* < .008), consistent by participants and by items (LRCS_1_ = 34.14, df = 33, *p* > .4; LRCS_2_ = 14.37, df = 15, *p* > .4). The probability of launching an eye movement towards where the glass had been was significantly higher in the ‘unmoved’ condition than in the ‘moved’ condition (LRCS_1_ = 7.39, df = 1, *p* < .008; LRCS_2_ = 4.40, df = 1, *p* < .04), consistent by participants and by items (LRCS_1_ = 25.30, df = 33, *p* > .8; LRCS_2_ = 11.26, df = 15, *p* > .7). Eye movements towards where the table had been were at the expense of eye movements towards where the glass had been, and vice versa (i.e. there was no effect of context on looks to non-critical regions: LRCS_1_ = 0.07, df = 1, *p* > .7; LRCS_2_ = 0.20, df = 1, *p* > .6), consistent by participants and by items (LRCS_1_ = 26.56, df = 33, *p* > .7; LRCS_2_ = 7.37, df = 15, *p* > .9). There were no more looks, collapsed across condition, to where the glass had been than towards where the table had been (LRCS = 0.14, df = 1, *p* > .7), consistent by participants and by items (LRCS_1_ = 31.29, df = 33, *p* > .5; LRCS_2_ = 12.69, df = 15, *p* > .6). That is, there was no residual bias towards one region or the other. To explore whether there was a residual tendency to move the eyes towards where the glass had actually been even in the ‘moved’ condition, we conducted further analyses which revealed that in this condition there were indeed slightly more saccades towards where the glass had been than towards the distractor region (LRCS = 4.28, df = 1, *p* = .04), consistent by participants and by items (both *p* > .2). Finally, looks to where the table had been in the ‘moved’ condition were as frequent as looks towards where the glass had been in the ‘unmoved’ condition (LRCS = 0.12, df = 1, *p* > .7; consistent by participants and by items – both *p* > .1).
(iii). *During ‘the glass’:* The probability of launching an eye movement towards the table region was significantly higher in the ‘moved’ condition than in the ‘unmoved’ condition (LRCS_1_ = 22.36, df = 1, *p* < .001; LRCS_2_ = 21.31, df = 1, *p* < .001), consistent by participants and by items (LRCS_1_ = 22.13, df = 33, *p* > .9; LRCS_2_ = 8.38, df = 15, *p* > .9). The probability of launching an eye movement towards the glass region was significantly higher in the ‘unmoved’ condition than in the ‘moved’ condition (LRCS_1_ = 25.09, df = 1, *p* < .001; LRCS_2_ = 15.82, df = 1, *p* < .001), consistent by participants and by items (LRCS_1_ = 21.43, df = 33, *p* > .9; LRCS_2_ = 13.03, df = 15, *p* > .6). Eye movements towards where the glass had been were at the expense of eye movements towards where the table had been, and vice versa (LRCS_1_ = 0.001, df = 1, *p* > .9; LRCS_2_ = 0.27, df = 1, *p* > .6), consistent by participants and items (LRCS_1_ = 24.37, df = 33, *p* > .8; LRCS_2_ = 8.33, df = 15, *p* > .9). There was no overall bias to look more towards where the glass had been than towards where the table had been (LRCS = 0.05, df = 1, *p* > .8), consistent by participants and items (LRCS_1_ = 15.27, df = 33, *p* > .9; LRCS_2_ = 8.78, df = 15, *p* > .8). The probability of making an eye movement in the ‘moved’ condition to where the glass had been did not differ from the probability of moving to the distractor region (LRCS = 0.29, df = 1, *p* > .5; consistent by participants and by items, both *p* > .4). Looks to where the table had been in the ‘moved’ condition were as frequent as looks towards where the glass had been in the ‘unmoved’ condition (LRCS = 0.27, df = 1, *p* > .6; consistent by participants and by items, both *p* > .5).
(iv). *At the offset of ‘the glass’:* the table region was fixated more at this point in time in the ‘moved’ condition than in the ‘unmoved’ condition (LRCS_1_ = 14.05, df = 1, *p* < .001; LRCS_2_ = 15.06, df = 1, *p* < .001), consistent by participants and by items (LRCS_1_ = 41.49, df = 33, *p* > .1; LRCS_2_ = 16.06, df = 15, *p* > .3). And conversely, the glass region was fixated more in the ‘unmoved’ condition than in the ‘moved’ condition (LRCS_1_ = 17.54, df = 1, *p* < .001; LRCS_2_ = 15.52, df = 1, *p* < .001), consistent by participants and by items (LRCS_1_ = 40.94, df = 33, *p* > .1; LRCS_2_ = 18.76, df = 15, *p* > .2). Increased fixations on where the table had been were at the expense of decreased fixations on where the glass had been and vice versa (LRCS_1_ = 0.01, df = 1, *p* > .9; LRCS_2_ = 0.04, df = 1, *p* > .8). This was consistent by participants and by items (LRCS_1_ = 31.83, df = 33, *p* > .5; LRCS_2_ = 10.77, df = 15, *p* > .7). There were no more fixations where the glass had been than where the table had been (LRCS = 2.57, df = 1, *p* > .1) consistent by participants and by items (LRCS_1_ = 42.85, df = 33, *p* > .1; LRCS_2_ = 21.43, df = 15, *p* > .1). In the ‘moved’ condition, the probability of fixating where the glass had been did not differ from the probability of fixating the distractor region (LRCS = 0.05, df = 1, *p* > .8; consistent by participants and by items, both *p* > .08). Fixations on the region where the table had been in the ‘moved’ condition were as frequent as fixations on the region where the glass had been in the ‘unmoved’ condition (LRCS = 0.98, df = 1, *p* > .3; consistent by participants and by items, both *p* > .2).

### 3.3 Discussion

The results from Experiment 2 are clear: there were statistically reliable effects of context on eye movements towards both where the table had been and where the glass had been, whether in respect of anticipatory saccades, concurrent saccades (i.e. during ‘*the glass*’) or fixations at the offset of the sentence-final ‘*glass*’. Moreover, these effects were symmetrical – the eyes were directed towards where the table had been in the ‘moved’ condition as often as they were directed towards where the glass had been in the ‘unmoved’ condition (and vice versa). Thus, the visual record of where the glass had been in the ‘unmoved’ condition was no more salient (in respect of attracting eye movements towards the corresponding location) than the linguistically induced event-based record of where the glass would be in the ‘moved’ condition. Moreover, in the ‘moved’ condition, the eyes were no more attracted during ‘*the glass*’ to where the glass had actually been than they were towards where the distractor had been. In other words, there was no residual bias in this condition to look towards the remembered location of the glass.^3^ Thus, the data from the ‘moved’ conditions indicate that the spatial representations that drove the eye movements in these studies were not reliant on objects actually having occupied particular locations within the scene. This is distinct from the situation described in Altmann (2004), in which anticipatory eye movements were observed towards a cake during ‘*eat*’ in ‘*The man will eat the cake*’ even though the corresponding scene had been removed prior to the onset of the spoken sentence. In Experiments 1 and 2, the glass had never occupied a position on or near the table, and yet its representation must have ‘inherited’, by means of the linguistic context, the spatial location associated with the table. We discuss below, in the general discussion, how this process might proceed.

After Experiment 1, we suggested that the stimulus-driven representation of the concurrent glass in the scene competed with the representation of that glass as instantiated in the event-representation constructed through the unfolding language. Our motivation for Experiment 2 was to eliminate this competition. This appears to have been accomplished, with no more looks towards where the actual glass had been located, in the ‘unmoved’ condition, than towards where the glass would be moved in the ‘moved’ condition; by eliminating the concurrent perceptual correlates of one of these representations (the glass that was on the floor), neither representation (the glass on the floor or on the table) was more salient than the other. Moreover, we would maintain that both representations were available to the cognitive system. We collected no evidence in this regard, but we do not believe that participants believed mistakenly, in the ‘moved’ condition, that the glass had originally been located on the table; participants more probably tracked the initial and end states of the glass, maintaining both representations as components of the moving event (indeed, the representation of that event entails the representation of both states). The likely availability of both representations is apparent in the following examples (which should be interpreted within the context of the visual scene depicted in Fig. 1):

(3) The woman will put the glass onto the table. Then, she will pick up the bottle, and pour the wine carefully into the glass.
(4) The woman will put the glass onto the table. But first, she will pick up the bottle, and pour the wine carefully into the glass.

Depending on the temporal connective ‘*then*’ or ‘*first*’, the glass into which the wine is poured is either located on the table (cf. 3) or on the floor (cf. 4). Further research is currently being undertaken to establish that participants’ eyes would return at the sentence-final ‘*glass*’ to the original location of the glass in (4) but to the new location on the table in (3); only two items in the current set of 16 used the ‘*but first*’ construction – too few to analyse separately. Nonetheless, the ease with which (4) can be comprehended (notwithstanding the difficulty induced by the mismatch between narrative and chronological order; cf. Mandler, 1986), including accommodation of the entailment that the glass is still on the floor, suggests that comprehenders can keep track of the distinct event-based representations of the glass and its locations.

In Experiment 2, neither of the event-based representations of the glass was accompanied, during the unfolding language, by the concurrent perceptual correlates of a glass. The representation of the glass on the floor was accompanied by *past* correlates (i.e. the visual memory of the glass), but equally, the representation of the glass on the table (as instantiated by the unfolding language) was accompanied by these same past perceptual correlates, to the extent that it was the same glass that had previously been seen (it was not some new glass). All that changed across the representations was that one representation included information about the floor-as-location, and the other included information about the table-as-location – and the fact that neither was accompanied by concurrent sensory stimulation resulted in each being equally salient (at least as defined operationally, in respect of both attracting eye movements in equal measure in the corresponding conditions). Thus, whereas in Experiment 2 these two representations competed on a level playing field, in Experiment 1 they did not.

## 4 General discussion

In the two studies reported above, linguistic contexts were used to manipulate the event-related locations of the objects that were portrayed in the concurrent scene. For example, participants fixated the table more often at the offset of ‘*glass*’ in ‘*she will pick up the bottle, and pour the wine carefully into the glass*’ when the preceding sentence had been ‘*The woman will put the glass onto the table*’ than when it had been ‘*The woman is too lazy to put the glass onto the table*’. Indeed, from ‘*pour*’ onwards, more saccadic eye movements were directed towards the table, or towards where the table had been, in the ‘moved’ condition than in the ‘unmoved’ condition. At first glance, these data suggest that overt visual attention is directed to particular locations that are, at least in part, determined by a dynamically modifiable representation of the objects’ locations, even when, as in Experiment 1, that representation is at odds with the location of the corresponding object in the concurrent visual scene.

Elsewhere, we and others (e.g. Altmann & Kamide, 2007; Chambers et al., 2004; Kamide et al., 2003) have stressed the importance of experientially-based knowledge in respect of the mapping between an unfolding sentence and the current visual world context. But the experientially-based knowledge we have of how an object interacts with its environment is just one source of information we access when interacting with an object. Crucially, it is the episodic, or situation-specific, knowledge associated with the individual experience of an object, in combination with knowledge abstracted over multiple previous experiences of such objects, that determines the mode of that interaction (i.e. how we might orient towards that object, how that object may impact on other specific objects in our immediate environment, and so on; see Chambers et al., 2004 for a demonstration of how possible modes of interaction with objects in the environment modulate language-mediated eye movements). The *location* of an object, such as the glass in the experiments reported here, is one aspect of the episodic experience associated with that object. So how is that represented, and how can the unfolding language influence the content of that representation? One view of visual cognition – situated vision – proposes that the encoding of the location of an object has an important function in respect of enabling the cognitive system to use the concurrent visual world as an aid to memory (cf. Ballard, Hayhoe, Pook, & Rao, 1997; O’Regan, 1992; Richardson & Spivey, 2000); activating an object’s ‘spatial pointer’ – an oculomotor coordinate defined relative to the configuration of cues within the scene – causes the eyes to move to the object’s location, enabling the retrieval of information about it that had perhaps not been encoded within the mental representation of that object. Depending on the task, it may be advantageous to store only minimal information about the object in that mental representation, thereby minimising memory load; if anything more needs to be recalled about that object, the spatial pointer can direct the eyes towards the object itself, at which time further information can be accessed directly from the visual percept. However, if the spatial pointer is simply a memory of some physical configuration of perceptual cues associated with the location of an object, that object must, at some time, have occupied a particular location. And yet, when the eyes fixated the ‘moved’ location of the glass in Experiments 1 and 2, the glass had not previously occupied that position. Does this mean that the spatial pointer associated with the representation of the glass need not have a sensory basis? In order to permit direct comparison between Experiments 1 and 2, the same stimuli (both visual and auditory) were used. In principle, however, the glass (and its equivalent across different stimuli) could have been removed from the scenes, and the auditory stimulus for the ‘moved’ condition changed to ‘*The woman will put a glass onto the table. Then, she will pick up the bottle, and pour the wine carefully into the glass*’. We would conjecture that the precise same pattern of eye movements would be found as in the ‘moved’ condition of Experiment 2. In this respect, the component of the mental representation of the glass that encoded its spatial location would not have a sensory (visual) basis.

According to Barsalou, Simmons, Barbey, and Wilson (2003), a sentence such as ‘The *woman will put the glass on the table*’ will engender a mental ‘simulation’ of the described event (a mental enactment of the experience of the event), in which case the spatial pointer corresponding to the eventual location of the glass in the ‘moved’ conditions of Experiments 1 and 2 might be considered a part of one such simulation. Although consideration of the relationship between ‘simulations’, ‘mental models’ (e.g. Johnson-Laird, 1983), and ‘situation models’ (e.g. van Dijk & Kintsch, 1983) is beyond the remit of this article, all three theoretical positions agree on the central role played by event representations – a role that is articulated most explicitly in versions of the event-indexing model (e.g. Zwaan, Langston, & Graesser, 1995; Zwaan & Radvansky, 1998). Within this general framework, the location to which the glass will move must be represented as part of the event structure constructed in response to the sentence that describes where, and when, the glass will be moved. And thus the representation of the glass’s future location is representationally (and *situationally*) distinct from its location within the concurrent or previous scene – the two representations have different experiential bases, with the actually experienced location based on perceptual properties of the configuration of objects within the scene, and the language-induced event-related location based on conceptual properties of the objects and their configuration within the scene. But given that both representations encode the configuration of objects within the scene, and that such configurational information, as distinct from absolute location, can form the basis for target-directed eye movements (cf. Spivey, Richardson, & Fitneva, 2004), both kinds of representation can support the targeting of saccadic eye movements.

There is an alternative account of why the eyes moved towards the table, when anticipating or hearing ‘*the glass*’ in the ‘moved’ conditions of Experiments 1 and 2. This alternative is not concerned with the spatial pointers associated with the representation of the glass, but rather with how the unfolding language might modify knowledge of the *table*. Our knowledge of a glass – its affordances – includes the fact that it can be drunk from, and that liquid can be poured into one. Our knowledge of a table is that it can, amongst other things, support objects. We might even suppose that this knowledge is probabilistic, with our knowledge of a table encoding the greater probability with which it may support a plate or a glass than a motorcycle (to this end, we would contend, for example, that a wine glass with a few drops of wine at the bottom would more likely be interpreted as having been fuller and subsequently drunk out of than as having been empty and subsequently filled with just those few drops – the latter is possible, albeit unlikely). In the experiments reported here, the linguistic context changed a number of things, including the future location of the glass, and the future situation-specific affordances of the table – namely, *that it would afford a glass with some more definite probability* (tables can always afford glasses or any other myriad number of objects, but which objects they afford at which times is situationally-determined). The notion here that the table could ‘afford’ a glass is no different from the notion that an empty wine glass could have afforded, in the past, some wine, or could afford, in the future, some wine. We have previously found (Altmann & Kamide, 2007) that such affordances mediate eye movements towards an empty wine glass or a full glass of beer as a function of whether participants hear ‘*the man will drink the…*’ or ‘*the man has drunk the…*’, with significantly more fixations on the empty wine glass at the onset of the postverbal determiner in the ‘*has drunk*’ condition than in the ‘*will drink*’ condition. Perhaps, in Experiments 1 and 2, participants fixated the table after ‘*glass*’, or indeed after ‘*pour*’ when anticipating an object into which something could be poured, because the knowledge they had of this table included the fact that, in the future, it would more definitely support a glass. In other words, the eyes moved towards the table because its affordances – knowledge of what it would hold in the future – matched the conceptual specification associated with the future tensed verb (see Altmann & Kamide, 2007, for an account of language-mediated eye movement control, based on such conceptual matching, which can be applied to both concurrent and ‘blank screen’ situations). Thus, we distinguish (as we did above) between affordances as knowledge abstracted across multiple experiences, and affordances as situation-specific knowledge that reflects the interaction between experience and the current situation.

One corollary of our approach is that, in the context of the ‘move the glass’ example, it matters that the woman moved the glass to a plausible location (that is, to a location that could plausibly afford the placement of a glass). But language is not limited to describing the plausible, or even the possible. Our claim that experientially-based event representations mediate our effects would predict that the eyes would not so readily move to the future location of the glass following a sentence such as ‘*The woman will put the glass on her head*’. A more perceptually-bound account, in which spatial location is encoded, and accessed, regardless of experientially-based event representations, would predict that the eyes would move to the new location as effortlessly when the glass was moved to the woman’s head as when it was moved to the table. Future research is required to rule out such an outcome.

Our data do not determine whether looks towards the table in the ‘moved’ conditions were due to location-specific knowledge associated with the future-event-based representation of the glass or were due to the future-situation-specific affordances of the table (and indeed, the two are not mutually exclusive; looks could have been due to a combination of both these sources of knowledge). Our data do reveal nonetheless that both the experiential and situationally-defined meaning of language interact with visual representations in determining where visual attention is directed as people understand language that refers to a visual scene. This is not particularly noteworthy, as it is unclear (from everyday experience) how cognition could function in any other way. What *is* noteworthy, we believe, is that our data reveal a dissociation that is possible between our representation of the currently experienced, or previously experienced, state of the (visual) world and other possible states, at other times, of that same world. In so doing, they reveal the manner in which eye movements reflect those same dissociations; the eye movements we have observed in these studies reflect a mental world whose contents appear, at least in part, to be dissociable from the concurrent, remembered, or imagined visual world, and it is *this* facet of our data that is novel. This dissociation, between the mental representations of objects and the perceptual correlates of those objects as depicted in a concurrent or prior scene, is due to the distinction between the sensory/perceptual experience of an object and the knowledge we have of that object. As suggested earlier, experientially-based encodings of the ways in which we interact with objects (and in which they interact with one another) require a representational substrate that encodes information that goes beyond that conveyed by the visual correlates of those objects. This experiential knowledge is critical in respect of causing attention to be attracted, in different circumstances, to certain objects more than to others. The nature of this knowledge speaks to the relationship between mental representations constructed on the basis of linguistic input on the one hand, and on the basis of visual scene processing on the other. We take the concept associated with an object in the real world to reflect, amongst other things (such as its physical form) the accumulated experience of the ways in which that object interacts with others in its real world environment — an idea that is mirrored in the language literature as the view that thematic roles reflect the accumulated experience of the events, and the entities that participate in those events, to which each verb in the language refers (McRae, Ferretti, & Amyote, 1997). In each case, the concept (whether associated with the object or with the verb-specific thematic role) is the accumulated experience of the interaction between one object and another. On this view, the *same* knowledge base underpins both the interpretation of the visual scene (in the absence of any language) and the interpretation of a sentence with reference to the verb’s thematic roles and the entities filling those roles (in the absence of a concurrent visual scene). In this respect, the visual scenes we have employed in our studies are simply a means to an end – they enable us to control the content of the mental representations within the context of which a particular sentence will be interpreted; the patterns of eye movements that accompany that interpretation enable us to probe the content of the representation that is being attended to as that interpretation develops in time.

Finally, our data suggest that theories of cognition (i.e. theories pertaining to the internal representation of external events) need to take account of the need for multiple representational instantiations of the same objects – instantiated with different event-specific properties. More specifically, they need to take account of the consequences of such multiple instantiations if, as we have suggested, they in fact compete with one another. Ellen Markman (personal communication) has suggested that the competition we have observed amongst multiple representational instantiations of the same object may even explain children’s poor performance on certain tasks such as the False Belief Task (Wimmer & Perner, 1983). In such tasks, the child must keep in mind multiple representations of the same object – the object starts off in one location, but is then moved to another, and this change in location is unseen by a protagonist whom the child is observing (or whom the child is being told about if the task is via story-telling). The child’s task is to say where the (deceived) protagonist thinks the object is (the correct answer corresponds to the original location, as the protagonist could not know that it had moved). The problem for the child is not so much that the object was in different locations before and after the movement, but rather that the child must represent both her own knowledge of the object’s location and the protagonist’s. Children aged 3-years will typically say that the protagonist thinks the object is in the *new* location. We conjecture that poor performance on such tasks may not reflect impoverished representation of beliefs *per se*, but may instead reflect competitive processes that favour one representation (the child’s actual knowledge) more than another (the protagonist’s presumed knowledge). Clements and Perner (1994) used evidence from children’s eye movements to argue that these distinct representations corresponding to the object at different locations do in fact co-exist in the traditional version of this task. A similar interpretation of the False Belief Task is given by Zaitchik (1990). She modified the task to show that performance in this task is unrelated to the child having to maintain a representation of the belief state of the protagonist. In her version of the task, a photograph was taken of the object before it was moved, and children were asked to say where, in the photograph, the object was located (the photograph was removed prior to the question). Children responded as if they had been asked where the protagonist thought the object was located – that is, they mistakenly reported the new location. Zaitchik argued that this behaviour arose because of the conflict between the child’s perceptual representation of the world *as it really was* and the child’s representation of the alternative state as represented in the photograph or the beliefs ascribed to the deceived protagonist. Our own proposal with respect to multiple representational instantiations of the same object is similar, although we place the burden of competition not at a propositional or situational level, but at the level of object representation. With respect to the transition from child to adult, it is conceivable that this involves a gradual shift in the weight given to the different features (perceptual, conceptual, temporal, and so on) which constitute the representational instantiations of each object. Our own data (Experiment 2) suggest that this shift results in an adult system which favours the perceptual correlates of the object-representations constructed through past perceptual experience no more than it does the conceptual correlates of the event-representations constructed through language.

The data reported here demonstrate how language can mediate the dynamic updating of a mental representation of a visual scene, and how this updated mental representation can form the basis for the subsequent direction of attention, irrespective of whether the scene is still present. These and other data lead us to believe that both anticipatory and concurrent eye movements reflect, in real-time, the unfolding interpretation of language with respect to a dynamically changing mental representation of a ‘real’ world to which that language may refer. It is this mental representation that guides behaviour. The challenge now is to understand how multiple instantiations of the same event-participants, reflecting the changes they undergo as the event unfolds, are distinguished within this medium.

## Figures and Tables

**Table 1:** Probabilities in Experiment 1 of fixating on, or launching saccades towards, the spatial regions occupied by the table or by the glass, calculated at the onset of the postverbal region (fixation analysis), during the postverbal region (saccadic analysis), during the sentence-final noun phrase (saccadic analysis), and at the offset of that noun phrase (fixation analysis). Numbers in parentheses indicate absolute number of trials on which a fixation on, or saccade to, each region was observed. For the saccadic analyses, the probabilities can sum to more than one because the eyes could saccade to more than one region in the available time. Equally, they can sum to less than one if no saccade was made during the interval of interest. Where the fixation probabilities sum to less than one, trials were lost through blinks, looks beyond the screen, or other failures to track the eye.

Analysis point/window	*…pour*∧*the wine carefully into the glass*		*…pour****the wine carefully into****the glass* (1246 ms)		*…pour the wine carefully into****the glass*** (541 ms)		*…pour the wine carefully into the glass*∧	
Analysis type	*p*(fixation)		*p*(saccade)		*p*(saccade)		*p*(fixation)	
Condition	Unmoved	Moved	Unmoved	Moved	Unmoved	Moved	Unmoved	Moved
Table	.04 (11)	.08 (21)	.13 (34)	.29 (73)	.06 (15)	.16 (40)	.04 (10)	.13 (32)
Glass	.11(29)	.11 (28)	.48 (123)	.44 (112)	.29 (74)	.25 (65)	.34 (88)	.27 (70)
Elsewhere	.74 (190)	.69 (176)	.79 (201)	.78 (200)	.50 (127)	.49 (125)	.47 (121)	.48 (122)

**Table 2:** Probabilities in Experiment 2 of fixating on, or launching saccades towards, the spatial regions corresponding to where the table, the glass, or the distractor had been, calculated at the onset of the postverbal region (fixation analysis), during the postverbal region (saccadic analysis), during the sentence-final noun phrase (saccadic analysis), and at the offset of that noun phrase (fixation analysis). Numbers in parentheses indicate absolute number of trials on which a fixation on, or saccade to, each region was observed. For each scene, the regions of interest corresponding to where the table, glass, or distractor had been were identically sized.

Analysis point/window	*…pour*∧*the wine carefully into the glass*		*…pour****the wine carefully into****the glass* (1246 ms)		*…pour the wine carefully into****the glass*** (541 ms)		*…pour the wine carefully into the glass*∧	
Analysis type	*p*(fixation)		*p*(saccade)		*p*(saccade)		*p*(fixation)	
Condition	Unmoved	Moved	Unmoved	Moved	Unmoved	Moved	Unmoved	Moved
Table	.06 (17)	.06 (15)	.07 (19)	.13 (36)	.02 (6)	.12 (32)	.07 (19)	.17 (46)
Glass	.08 (22)	.06 (15)	.14 (39)	.07 (20)	.10 (28)	.03 (8)	.14 (37)	.04 (11)
Distractor	.06 (15)	.04 (11)	.05 (14)	.03 (9)	.02 (6)	.02 (6)	.06 (16)	.04 (10)
Elsewhere	.61 (166)	.68 (185)	.27 (73)	.24 (64)	.13 (34)	.15 (40)	.56 (153)	.58 (157)

## Appendix 1

The sentential stimuli used in Experiments 1 and 2. There were two versions of each item, corresponding to the ‘moved’ and ‘unmoved’ conditions. Also shown is a list of the objects in the corresponding scene; the final object in the list corresponds to the distractor in Experiment 2.

The woman will put the glass onto the table. Then, she will pick up the bottle, and pour the wine carefully into the glass. [‘moved’ condition].
The woman is too lazy to put the glass onto the table. Instead, she will pick up the bottle, and pour the wine carefully into the glass. [‘unmoved’ condition].
[*woman, table, bottle of wine, empty wine glass, bookcase*].

The woman will put the bread onto the plate. Then, she will take some butter, and spread it sluggishly onto the bread.
The woman decided not to put the bread onto the plate. She will take some butter, and spread it sluggishly onto the bread.
[*woman, worktop, empty plate, butter dish with butter, slice of bread on board, coffee cup*].

The office worker will drag the dustbin right next to the fan. Then, he will grab the can, and chuck it violently into the dustbin.
The office worker has just dragged the dustbin away from the fan. Now, he will grab the can, and chuck it violently into the dustbin.
[*man at desk, floor fan, drinks can, wastebin, swivel chair*. The fan was on the far left of the scene, to ensure that the region of interest corresponding to ‘next to the fan’ was a constrained region to just one side].

The woman will lift the pet carrier onto the table. Then, she will take hold of the cat, and put it carefully into the pet carrier.
The woman has just lifted the pet carrier down from the table. Now, she will take hold of the cat, and put it carefully into the pet carrier.
[*woman, table, cat, pet carrier, picture*].

The businessman will put the computer onto the desk. Then, he will pick up the disk, and insert it gently into the computer.
The businessman was unable to put the computer onto the desk. But, he will pick up the disk, and insert it gently into the computer.
[*man, desk, computer disk on floor, computer, briefcase*].

The secretary will move the folder right next to the lamp. Then, she will look at the documents, and file them efficiently in the folder.
The secretary has moved the folder away from the lamp. She will look at the documents, and file them efficiently in the folder.
[*woman, desktop, desk lamp, pile of documents, document folder, ink stamp*. The lamp was on the far right of the scene, to ensure that the region of interest corresponding to ‘next to the lamp’ was a constrained region to just one side].

The woman will place the pan onto the cooker. Then, she will reach for the bowl, and transfer the eggs swiftly into the pan.
The woman will soon place the pan onto the cooker. But first, she will reach for the bowl, and transfer the eggs swiftly into the pan.
[*woman, worktop, cooker, bowl with eggs, frying pan, pepper mill*].

The housewife will move the vase onto the sideboard. Then, she will pick up the flowers, and arrange them delicately in the vase.
The housewife is too tired to move the vase onto the sideboard. But, she will pick up the flowers, and arrange them delicately in the vase.
[*woman, sideboard, flowers on floor, vase, books*]

The chef will take the pan to the cooker. Then, he will notice the lid, and place it quickly onto the pan.
The chef will check the pan and the cooker. Then, he will notice the lid, and place it quickly onto the pan.
[*chef, worktop, cooker, pan lid, pan with vegetables, knife*].

The woman will slide the jewellery box right next to the coffee. Then, she will admire the necklace, and hide it quickly inside the jewellery box.
The woman will examine the jewellery box as she drinks the coffee. Then, she will admire the necklace, and hide it quickly inside the jewellery box.
[*woman at table, coffee cup, necklace, jewellery box, door.* The smaller objects were arranged on the table top so that a constrained region could be defined corresponding to ‘next to the coffee’].

The man will shift the box onto the worktop. Then, he will lift up the pizza, and put it carefully into the box.
The man has just shifted the box off the worktop. Now, he will lift up the pizza, and put it carefully into the box.
[*man, worktop, napkin, fork, pizza, empty pizza box, fridge*].

The man will put the gramophone onto the sideboard. Then, he will clean the record, and place it carefully on the gramophone.
The man will soon put the gramophone onto the sideboard. But first, he will clean the record, and place it carefully on the gramophone.
[*man in chair, sideboard, record, gramophone player, bongos*].

The girl will suspend the hanger on the rail. Then, she will reach for the shirt and hang it cheerfully onto the hanger.
The girl has taken the hanger off the rail. Now, she will reach for the shirt and hang it cheerfully onto the hanger.
[*girl, clothes rail, shirt on chair, coat hanger, plant*].

The man will move the cup onto the table. Then, he will reach for the tea pot, and pour the tea slowly into the cup.
The man has taken the cup off the table. Now, he will reach for the tea pot, and pour the tea slowly into the cup.
[*man, table, teapot, tea cup, chair*].

The man will drag the chair over to the girl. Then, he will lift up the teddy bear, and sit it affectionately on the chair.
The man will look at the chair and then at the girl. Then, he will lift up the teddy bear, and sit it affectionately on the chair.
[*man, girl, teddy bear, chair, Christmas tree.* The girl was on the far left of the scene, to ensure that the region of interest corresponding to ‘over to the girl’ was a constrained region to just one side].

The woman will move the mug onto the trolley. Then, she will reach for the bottle, and tip the water quickly into the mug.
The woman has taken the mug off the trolley. Now, she will reach for the bottle, and tip the water quickly into the mug.
[*woman, trolley, bottle of water, mug on table, dustbin*].

## Footnotes

1 A significant 2-way interaction between condition and object in the LRCS_1_ and LRCS_2_ analyses would indicate generalizability across participants and items respectively. In addition, the lack of a 3-way interaction (condition × object × participants/items) would indicate consistency in the magnitude of the effect of condition across participants or items. For ease of exposition, we describe an effect as ‘more looks towards A in condition *X* than in condition *Y*’ when we are in fact referring to the interaction between condition (*X* vs. *Y*) and object (*A* vs. *B*), and we describe an effect as ‘consistent’ by participants or items when we are in fact referring to the 3-way interaction with either participants or items. We used SPSS Version 16 to analyse these data; within SPSS non-interaction effects (e.g. the absolute difference in looks towards *A* vs. looks towards *B*) do not yield different values of LRCS by participant or by item, although higher-order interactions with such effects (as would be tested in order to determine consistency in the magnitude of the difference) do yield different values. The same is not true of other statistical packages (e.g. Statistica), which produce partial association main effect values of LRCS that may differ between participant and item analyses. The difference appears to be due to the ways in which these packages derive expected frequencies for main effects (Christoph Scheepers, pers. comm.)

2 A version of Experiment 1 was run separately in which we contrasted, for the ‘moved’ condition alone, the tense of each sentence: In the ‘future’ condition, the sentences were as in Experiment 1, and in the ‘past’ condition they were changed to the past tense (e.g. ‘*The woman put the glass onto the table. Then, she picked up the bottle, and poured the wine carefully into the glass.*’) There was no effect of tense on looks towards either the glass or the table. As will become clear in the main text, the critical issue is not tense in these cases, but the requirement to maintain distinct representations of the state of the glass at different moments in event-time. Nonetheless, other studies have demonstrated that tense does have an important role with respect to interpreting the scene as depicting the initial or final state in the described event (Altmann & Kamide, 2007).

3 Further inspection of the data revealed that the slight tendency to look towards the original location of the glass in the ‘moved’ condition during the underlined interval ‘*pour the wine carefully into the glass*’ (see Table 2) was due to a small increase in saccades towards this location during ‘*carefully into*’ (there was no such increase during ‘*the wine*’). Hence, there was some small residual attraction of the location originally occupied by the glass that did influence anticipatory eye movements. Crucially, the location originally occupied by the table was significantly more attractive, nonetheless.

## References

[bib1] Allopenna P.D., Magnuson J.S., Tanenhaus M.K. (1998). Tracking the time course of spoken word recognition using eye movements: Evidence for continuous mapping models. Journal of Memory and Language.

[bib2] Altmann G.T.M. (2004). Language-mediated eye movements in the absence of a visual world: The ‘blank screen’ paradigm. Cognition.

[bib3] Altmann G.T.M., Kamide Y. (1999). Incremental interpretation at verbs: Restricting the domain of subsequent reference. Cognition.

[bib4] Altmann G.T.M., Kamide Y., Ferreira F. (2004). Now you see it, now you don’t: Mediating the mapping between language and the visual world. The integration of language, vision, and action: Eye movements and the visual world.

[bib5] Altmann G.T.M., Kamide Y. (2007). The real-time mediation of visual attention by language and world knowledge: Linking anticipatory (and other) eye movements to linguistic processing. Journal of Memory and Language.

[bib6] Ballard D.H., Hayhoe M.M., Pook P.K., Rao R.P.N. (1997). Deictic codes for the embodiment of cognition. Behavioural and Brain Sciences.

[bib7] Barsalou L.W., Simmons W.K., Barbey A.K., Wilson C.D. (2003). Grounding conceptual knowledge in modality-specific systems. Trends in Cognitive Sciences.

[bib8] Chambers C.G., Tanenhaus M.K., Magnuson J.S. (2004). Actions and affordances in syntactic ambiguity resolution. Journal of Experimental Psychology: Learning, Memory and Cognition.

[bib9] Clements W.A., Perner J. (1994). Implicit understanding of belief. Cognitive Development.

[bib10] Cooper R.M. (1974). The control of eye fixation by the meaning of spoken language: A new methodology for the real-time investigation of speech perception, memory, and language processing. Cognitive Psychology.

[bib11] Dahan D., Magnuson J.S., Tanenhaus M.K., Hogan E.M. (2001). Subcategorical mismatches and the time course of lexical access: Evidence for lexical competition. Language and Cognitive Processes.

[bib12] Dowty D. (1979). Word meaning and Montague grammar.

[bib13] Ferreira F., Ferraro V., Bailey K.G.D. (2002). Good-enough representations in language comprehension. Current Directions in Psychological Science.

[bib14] Gibson J.J., Shaw R.E., Bransford J. (1977). The theory of affordances. Perceiving, acting, and knowing.

[bib15] Henderson J.M., Ferreira F., Henderson J.M., Ferreira F. (2004). Scene perception for psycholinguists. The interface of language, vision and action.

[bib1a] Hommel B., Müsseler J., Aschersleben G., Prinz W. (2001). The theory of event coding (TEC): A framework for perception and action planning. Behavioral and Brain Sciences.

[bib16] Hoover M.A., Richardson D.C. (2008). When facts go down the rabbit hole: Contrasting features and objecthood as indexes to memory. Cognition.

[bib17] Huettig F., Altmann G.T.M. (2005). Word meaning and the control of eye fixation: Semantic competitor effects and the visual world paradigm. Cognition.

[bib18] Johnson-Laird P.N. (1983). Mental models: Towards a cognitive science of language, inference, and consciousness.

[bib19] Kamide Y., Altmann G.T.M., Haywood S.L. (2003). The time-course of prediction in incremental sentence processing: Evidence from anticipatory eye movements. Journal of Memory and Language.

[bib20] Knoeferle P., Crocker M.W. (2006). The coordinated interplay of scene, utterance, and world knowledge: Evidence from eye tracking. Cognitive Science.

[bib21] Knoeferle P., Crocker M.W. (2007). The influence of recent scene events on spoken comprehension: Evidence from eye movements. Journal of Memory and Language.

[bib22] Knoeferle P., Crocker M.W., Scheepers C., Pickering M.J. (2005). The influence of the immediate visual context on incremental thematic role-assignment: Evidence from eye-movements in depicted events. Cognition.

[bib23] Mandler J.M. (1986). On the comprehension of temporal order. Language and Cognitive Processes.

[bib24] McRae K., Ferretti T.R., Amyote L. (1997). Thematic roles as verb-specific concepts. Language and Cognitive Processes.

[bib25] O’Regan J.K. (1992). Solving the ‘real’ mysteries of visual perception: The world as an outside memory. Canadian Journal of Psychology.

[bib26] Richardson D.C., Spivey M.J. (2000). Representation, space and Hollywood squares: Looking at things that aren’t there anymore. Cognition.

[bib27] Scheepers C. (2003). Syntactic priming of relative clause attachments: Persistence of structural configuration in sentence production. Cognition.

[bib28] Spivey M.J., Richardson D.C., Fitneva S.A., Henderson J.M., Ferreira F. (2004). Thinking outside the brain: Spatial indices to visual and linguistic information. The interface of language, vision, and action: Eye movements and the visual world.

[bib29] Steedman M.J. (2002). Plans, affordances, and combinatory grammar. Linguistics and Philosophy.

[bib30] Tanenhaus M.K., Spivey-Knowlton M.J., Eberhard K.M., Sedivy J.C. (1995). Integration of visual and linguistic information in spoken language comprehension. Science.

[bib31] van Dijk T.A., Kintsch W. (1983). Strategies in discourse comprehension.

[bib32] Wimmer H., Perner J. (1983). Beliefs about beliefs: Representation and constraining function of wrong beliefs in Young children’s understanding of deception. Cognition.

[bib33] Zaitchik D. (1990). When representations conflict with reality: The preschooler’s problem with false beliefs and “false” photographs. Cognition.

[bib34] Zwaan R.A., Langston M.C., Graesser A.C. (1995). The construction of situation models in narrative comprehension: An event-in-dexing model. Psychological Science.

[bib35] Zwaan R.A., Radvansky G.A. (1998). Situation models in language comprehension and memory. Psychological Bulletin.

